# Teleost innate immunity, an intricate game between immune cells and parasites of fish organs: who wins, who loses

**DOI:** 10.3389/fimmu.2023.1250835

**Published:** 2023-10-16

**Authors:** Bahram Sayyaf Dezfuli, Massimo Lorenzoni, Antonella Carosi, Luisa Giari, Giampaolo Bosi

**Affiliations:** ^1^ Department of Life Sciences and Biotechnology, University of Ferrara, Ferrara, Italy; ^2^ Department of Chemistry, Biology and Biotechnologies, University of Perugia, Perugia, Italy; ^3^ Department of Environmental and Prevention Sciences, University of Ferrara, Ferrara, Italy; ^4^ Department of Veterinary Medicine and Animal Science, University of Milan, Lodi, Italy

**Keywords:** immune cells, macrophages, neutrophils, mucous cells, mast cells, rodlet cells, metazoan parasites, teleost

## Abstract

Fish, comprising over 27,000 species, represent the oldest vertebrate group and possess both innate and adaptive immune systems. The susceptibility of most wild fish to parasitic infections and related diseases is well-established. Among all vertebrates, the digestive tract creates a remarkably favorable and nutrient-rich environment, which, in turn, renders it susceptible to microparasites and macroparasites. Consequently, metazoan parasites emerge as important disease agents, impacting both wild and farmed fish and resulting in substantial economic losses. Given their status as pathogenic organisms, these parasites warrant considerable attention. Helminths, a general term encompassing worms, constitute one of the most important groups of metazoan parasites in fish. This group includes various species of platyhelminthes (digeneans, cestodes), nematodes, and acanthocephalans. In addition, myxozoans, microscopic metazoan endoparasites, are found in water-dwelling invertebrates and vertebrate hosts. It is worth noting that several innate immune cells within the fish alimentary canal and certain visceral organs (e.g., liver, spleen, and gonads) play active roles in the immune response against parasites. These immune cells include macrophages, neutrophils, rodlet cells, and mast cells also known as eosinophilic granular cells. At the site of intestinal infection, helminths often impact mucous cells number and alter mucus composition. This paper presents an overview of the state of the art on the occurrence and characteristics of innate immune cells in the digestive tract and other visceral organs in different fish-parasite systems. The data, coming especially from studies employed immunohistochemical, histopathological, and ultrastructural analyses, provide evidence supporting the involvement of teleost innate immune cells in modulating inflammatory responses to metazoan and protozoan parasitic infections.

## 1 Introduction

In vertebrates, the immune system has evolved to discriminate between self (host tissue) and non-self (pathogens). It consists of two components: the innate system and the adaptive system (1). While all animals possess an innate immune system, the adaptive immune system develops later, appearing first in *Gnathostomata* or jawed vertebrates (2). Fish, as the first vertebrate class to possess both types of immune systems, serve as a crucial model for investigating the evolutionary history of immune systems in vertebrates and comparative immunology (3, 4). The innate immune system, which comprises epithelial/mucosal barriers, humoral parameters, and immune cells, is the initial responder to infection, it plays a pivotal role in disease resistance and has a kind of memory called trained innate immunity which differs from adaptive memory for many aspects (2, 5).

In fish, the gills, skin, and gut act as mucosal barriers, serving as the first line of defense. These dynamic structures enable the animal to interact with the surrounding environment while maintaining homeostasis (6). Fish mucosal barriers possess several important properties. Firstly, they contain immune cells and effector molecules within their anatomical structures. Additionally, the mucus layer acts as a physical barrier and contains potent bioactive molecules (7). Recent studies have provided insights into the features of fish mucosal immunity and its roles in exposure to contaminants, stress, vaccination, wound repair, and infection (8). In teleosts, the intestinal mucosa holds particular immunological significance as it interacts with leukocyte subpopulations that mediate both adaptive and innate immune responses (9).

Intestinal parasites induce alterations in the structure of the gut tissue, which in turn affect its normal function (10). Enteric worms commonly induce gut inflammation and elicit host immune reactions (11–13). Inflammation is a complex series of homeostatic mechanisms involving the nervous, circulatory, and immune systems in response to organ injury or infection (12, 14). If the acute inflammatory response fails to eliminate the pathogen, the inflammatory process persists and acquires new characteristics (15).

Studies on fish have reported the essential role of enteric neuromodulators and the immune system in the inflammatory process caused by endoparasites (16–18). The relationship between mucous cells and neuroendocrine cells in fish harboring intestinal helminths has been described in previous studies (19, 20). Enteric parasites commonly enhance the secretion of mucous cells (21, 22). Fish mucus is involved in excretion, feeding, respiration, reproduction, ionic and osmotic regulation, and protection against parasites (22). In some fish species, mucous cells have been found to produce and release defense substances such as antimicrobial peptides (AMPs) (23). Mucous cells are components of the innate immune system (1, 24).

In addition to mucous cells, various cell types contribute to the innate immunity of teleost fish. These include granulocytes, such as mast cells (MCs), neutrophils, monocytes/macrophages, and rodlet cells (RCs) (12, 17, 25), as well as non-specific cytotoxic cells and natural killer-like cells (1). Our aim is to highlight exciting new advances in our understanding of fish immune mechanisms against enteric parasites and worms that infect visceral organs.

## 2 Actors in fish innate immunity and their responses against parasites

In this section, we will sequentially examine the major types of innate immune cells.

### 2.1 Mucous cells

Within the gut, mucous epithelial cells, also known as goblet cells, are responsible for mucus production and its holocrin secretion on the epithelial surface (20). Mucus plays a critical role in mucosal defense mechanisms (26–29), and mucous cells are considered a specific type of innate immune cell (24, 28). Mucous cells exhibit a basal elongated nucleus and possess supranuclear spherical or polyhedric vacuoles that contain more or less mucus, depending on the cell’s maturation stage. At the ultrastructural level, the mucus within the vacuoles may appear electron-opaque or, in certain cases, electron-lucent (20).

Gastrointestinal mucus has long been regarded as a lubricant that aids in the transit of digesta and protects the gut mucosa from mechanical damage (22, 30). Numerous studies have demonstrated that the chemical composition of mucus varies across different regions of the digestive tract and depending on its physiological state (25, 30–32). During stress or inflammation caused by pathogenic organisms, carboxylate and sulfate acidic mucus components increase (22, 25). Thomsson et al. (33) reported rapid glycosylation changes in the mucus of the intestine of rainbow trout *Oncorhynchus mykiss* infected with *Aeromonas hydrophila* and *A. salmonicida*.

Mucins, which are the primary components of vertebrate mucus, are high molecular weight proteins consisting of long peptide chains adorned with hundreds of O-linked oligosaccharides (27). The expression of mucins changes in the presence of enteric infections and varies depending on the type of pathogen (27). Limited information is available regarding the effects of intestinal parasites on differential mucin expression in fish. Perez-Sanchez et al. (27) reported the downregulation of three mucins (Muc13, Muc2, and Muc2-like) in the intestines of gilthead seabream, *Sparus aurata*, infected with the myxozoan *Enteromyxus scophthalmi*. Furthermore, myxozoan infection elicited higher glycosylation levels in the gut mucus composition of gilthead seabream, reducing pathogen adhesion (34). Schroers et al. (35) demonstrated changes in mucus composition in the gut of the common carp, *Cyprinus carpio*, following per oral treatment with the bacterium *Aeromonas hydrophyla*. During the infection, mucosal adhesion of pathogen is an essential initial step (36, 37). In infected fish, alterations in the glycosylation patterns of intestinal mucus serve as a mechanism to hinder pathogens adhesion to the epithelial surface and the activity of their enzymatic complexes (38).

An increase in the total number of mucous cells was observed in the intestines of fish infected with helminths, particularly near their attachment sites (19, 20, 22, 39). The hyperplastic response of intestinal mucous cells to helminth infections has been reported in various parasite-fish systems, such as *Salmo trutta* and *Squalius cephalus* infected with *Pomphorhynchus laevis* (Acanthocephala) (20, 39, 40), *Salmo trutta* infected with *Echinorhynchus truttae* (Acanthocephala) or *Cyathocephalus truncatus* (Cestoda) (20, 41), *Anguilla anguilla* infected with *Acanthocephalus rhinensis* (Acanthocephala) or *Helicometra fasciata* (Trematoda) (20), and *Tinca tinca* infected with *Monobothrium wageneri* (Cestoda) (20). Intestinal helminths induce the secretion of abundant mucus into the lumen (19, 20, 25, 39–42). Lectin histochemistry revealed remarkable changes in the mucus oligosaccharides of mullet intestines infected with *Neoechinorhynchus agilis* (Acanthocephala) compared to uninfected conspecifics (22). These changes include increased mucus viscosity due to higher amounts of sulfated mucins, providing resistance to degradation by bacterial lytic enzymes (22, 43, 44). Infected fish exhibit mucins that are rich in terminal sialic acid residues, which inhibit bacterial adhesion to the epithelial surface (22, 35).

The mucous cells of fish intestines are believed to play a role in the secretion of AMPs like piscidins (45–52), and peptides such as inducible nitric oxide synthase (i-NOS) (53). In the broad gilled hagfish *Eptatretus cirrhatus*, an ancient jawless fish, mucous cells in the intestinal tract were identified through the use of antibodies targeting the biogenic amine serotonin, Toll-Like Receptor 2 (TLR-2), piscidin1, and i-NOS (54). In their record, Alesci et al. (54) mentioned the co-occurrence of serotonin/TLR-2, and i-NOS/piscidin1 in the *E. cirrhatus* intestinal mucous cells, using the “colocalization view” with the software Zen 2011. Nevertheless, a further independent confirmation might be necessary (i.e. analysis of the Pearson’s coefficient, 55). Additionally, the same authors highlighted the presence of immunoreactivity to the anti-vesicular acetylcholine transporter (VAChT) antibody in mucous cells of the intestines of *Heteropneustes fossilis* and *Heterotis niloticus*, indicating the ectopic presence of acetylcholine (56). Acetylcholine, which controls various vital cellular functions (e.g. proliferation, differentiation, establishment and maintenance of cell-cell contacts), is secreted by several non-neuronal cells (57). Despite the relatively limited observations made by the aforementioned authors, there have been no definitive studies establishing the ability of fish mucous cells to produce serotonin and i-NOS/piscidins. Therefore, the results of their research are yet to be confirmed.

In the intestines of infected fish, cholinergic signals play a role in mucus secretion and discharge (19, 58). For example, in chub intestines parasitized with *P. laevis*, close proximity between endocrine epithelial cells secreting galanin, serotonin, and enkephalins and mucous cells has been observed. This indicates a strong association between paracrine signals from endocrine cells and mucus discharge (19). Furthermore, in the same host-helminth system, the use of confocal and transmission electron microscopies has revealed the presence of mast cells (MCs) in the vicinity of intestinal mucous cells and often MCs were found in degranulation (25, 59). Similarly, intraepithelial MCs adjacent to mucous cells have been documented in the intestine of *Silurus glanis* infected with the cestode *Glanitaenia osculata* (16). Many researchers concur that the degranulation of MCs and the heightened production of mucus in the vertebrate gut are part of a defensive mechanism against intestinal parasites (16, 25, 60–63).

With reference to mammals and intestinal helminths, excessive mucus secretion has been suggested to aid in the removal of worms from the gut lumen (64). Several studies have focused on the hyperplasia of mucous cells, resulting in an increase in mucus secretion in various fish-helminth systems (19, 41). We have documented an elevated density of mucous cells in the fish gut and observed qualitative changes in the glycoconjugates secreted in response to helminths (12, 19, 22, 25). Accumulating evidence suggests that mucus secretion primarily functions to protect the underlying mucosa from worm mechanical damage and invasion by pathogenic microorganisms (12, 22, 25, 35, 40, 41).

### 2.2 Mast cells

Mast cells (MCs) are crucial components of the host defense system (65). Mast cells are secretory cells that have been conserved for over 500 million years in all vertebrate classes, predating the development of adaptive immunity (66). While MCs comprise a heterogeneous cell population, they serve as initiators and effectors of innate immunity and regulators of the adaptive immune response. Across all vertebrates, they share similar morphology and function (67). In mammals, MCs are critical for controlling the bacteria burden (68). Recent literature by Dahlin et al. (69) provides a comprehensive review of MC behavior and function in mammals. In fish, the acidic and basic contents of MC cytoplasmic granules vary among species and often exhibit different metachromasia based on the staining method used (65, 70). Fish MCs display an irregular shape, eccentric nucleus, and numerous electron-dense cytoplasmic granules (63).

MCs are commonly found in connective tissues of most fish species. They are primarily located inside or in close proximity to the blood vessels of the gill and mucosal layer of the intestine. This particular positioning enables MCs to fulfill a crucial role in host defense (12, 18, 65, 71). Within mucosae, MCs frequently coexist with other innate immune cells such as neutrophils, mucous cells, rodlet cells, and macrophage aggregates (22, 59). In certain species, they can also be found in the intraepithelial position (16, 22), liver (72, 73), and gonad (74).

At the site of inflammation and in the presence of damaged tissue, MCs release a range of inflammatory mediators, including several proteolytic enzymes, cytokines, arachidonic acid metabolites and piscidins (45–47). Piscidins exhibit potent, broad-spectrum antimicrobial activity against viruses, bacteria, fungi, and metazoan parasites (47–50). Molecular analyses of piscidins in different fish species have revealed high variability in length and amino acid sequence (50, 51). Piscidins 3 and 4 have been detected in the intestinal MCs of hybrid striped bass (*Morone saxatilis* × *M. chrysops*) (45) and gilthead seabream (46, 52, 75). However, piscidins 3 and 4 were absent in the intestines of barbel and wels catfish infected with the acanthocephalan *Pomphorhynchus laevis* (76), providing further evidence of the distinct taxonomic distributions of piscidins (45, 46, 52). In the medium intestine of the goldfish *Carassius auratus*, MCs exhibit immunoreactivity to antibodies against TLR-2 and S100 (77). TLR-2 is an antimicrobial peptide receptor that recognizes gram-positive bacteria (78). Detection of pathogen molecules by TLR-2 triggers the activation of macrophages and dendritic cells, leading to cytokine secretion (79, 80). S100 is a peptide with antimicrobial activity that has been detected in various types of immune cells, including neutrophils, monocytes/macrophages, and MCs (77, 81–83). Mast cells might contain histamine (67, 84), serotonin (77, 85, 86), Tumor Necrosis Factor-α (TNF-α) (87), and mucopolysaccharides with residues of α-N-acetyl-galactosamine (59). 

Mast cells frequently respond to parasites by undergoing degranulation, releasing their contents. This process has been observed in fish infected with metazoans (25, 88). In the intestine of brown trout infected with the cestode *Cyathocephalus truncatus* and acanthocephalans *Echinorhynchus truttae* and *Dentitruncus truttae*, the migration and accumulation of MCs at the site of parasitic infection have been observed in large numbers (71, 89, 90). A similar finding was observed in the gut of powan-infected with the cestode *Diphyllobothrium dendriticum* (91). In wels catfish, *Silurus glanis* parasitized by the cestode *Glanitaenia osculata*, a high number of MCs were observed in the medium intestine compared to uninfected conspecifics and MCs were often observed in close proximity to endocrine epithelial cells (16). Furthermore, parasitized wels catfish exhibited a higher number of endocrine epithelial cells immunoreactive to met-enkephalin, galanin, and serotonin (16). Endocrine epithelial cells are part of the gut neuroendocrine system and interact with and cooperate with immune cells in response to helminths (16, 19) and pathogens or inflammation caused by them (92–94). Remarkably, extraintestinal infections in *Gasterosteus aculeatus* by larvae of *P. laevis* have been documented, with MCs found on the surface of the worm, and granules penetrating the tegument of the parasite (95).

### 2.3 Neutrophils

Neutrophils are among the first cell types to arrive at the site of tissue injury or infection (96, 97). Neutrophils exhibit a round to oval shape with an irregular outline and a lobed nucleus (73). Cytoplasm of neutrophils contains smaller granules compared to those of MCs. These granules have a rod-shaped structure and possess an elongated electron-dense lamellar core (72). Unlike mammals, where neutrophils represent the predominant leukocytes during homeostasis, in fish neutrophils account for approximately 5% of circulating leukocytes (98). Kidney of teleost as hematopoietic organ has the largest population of neutrophils, which can be rapidly mobilized through blood vessels to sites of inflammation (98, 99). They are guided to the target site by chemotactic signals (99). In fish as in mammals, the chemokine interleukin-8 (IL-8, also known as CXCL8) is involved in recruiting neutrophils to the site of inflammation (100, 101). These highly motile cells play a crucial role in the initial defense through phagocytosis of microbes, secretion of granule proteins, and release of other antimicrobials (102, 103). The plasmalemma of neutrophils contains antimicrobial peptide receptors that directly bind to pathogenic microorganisms, facilitating their engulfment and internalization within the cytoplasmic phagosome. Subsequently, the phagosome fuses with a lysosomal vacuole (104). In addition to phagocytosis, neutrophils secrete active molecules and radicals such as nitric oxide, reactive oxygen species, and reactive nitrogen species (105). These reactive substances exert biocidal actions against bacteria and parasites, and emerging evidence suggests their involvement in cytokine responses and modulation of immune cell apoptosis (106). Studies on zebrafish have shown that neutrophils do not always undergo apoptosis during inflammation resolution but can often migrate from damaged tissues back to the vasculature. This process, known as reverse transmigration, is regulated by retrograde chemotaxis (107, 108). The cytoplasmic granules of neutrophils contain mainly myeloperoxidase, a highly cationic glycosylated enzyme primarily produced by these leucocytes (109, 110). Neutrophils also contribute to proinflammatory responses by releasing cytokines that activate and recruit other host immune cells (103).

At the site of inflammation, neutrophils recruited to the area release extracellular traps (NETs), which consist of smooth chromatin fibers combined with histones and granule components (99, 111). NETs immobilize and reduce the virulence of extracellular micropathogens, preventing their dissemination and facilitating their elimination (99, 110–112). Additionally, NETs help maintain a high local concentration of antimicrobial peptides found in degranulated neutrophils (111).

Neutrophils interact with various aquatic pathogens, including fish virus (113), Gram-negative bacteria (114, 115), protozoans (97, 116, 117), flatworm monogeneans (118), and digeneans (119). In the case of other helminths, in the intestine of the tench parasitized with the cestode *Monobothrium wageneri*, numerous neutrophils in degranulation were observed in close proximity to the microtriches of the worm (120). Neutrophils have also been documented to be in close proximity to the nematode body (121) and encysted nematode larvae in the pancreas and liver of the minnow (72). The relationship between neutrophils and aquatic pathogens has been recently reviewed by Buchmann (104). It has been documented that neutrophils have various functions in both adaptive and innate immunity, including proinflammatory roles. However, their contributions to the resolution of inflammation have been limited to apoptotic cell death and subsequent clearance by macrophages (103, 122).

### 2.4 Macrophages

The primary phagocytic cells in vertebrates are macrophages and their precursor monocytes. In response to tissue injury or infection caused by parasitic pathogens, monocytes are promptly recruited and undergo differentiation into tissue macrophages (123). Similar to other vertebrates, cells of the macrophage lineage contribute to the immune responses in fish. Consequently, recent studies in fish immunology have specifically targeted these cells (124).

Fish macrophages are found throughout the body cavity and various organs, including kidney, spleen, intestine, liver, and gills (109). Macrophages are characterized as large cells with an irregular outline, containing vesicular structures with electron-lucent vesicles and electron-opaque contents (76). Macrophages often contain pigments like hemosiderin, lipofuscin, and melanin (109) and can be organized in groups known as melano-macrophage centers or macrophage aggregates (MAs) (125, 126).

Recent studies have reported the presence of resident macrophage populations in various tissues, which exhibit rapid and highly specific responses to pathogen-induced damage (127). The precise mechanisms by which resident macrophages contribute to development, tissue homeostasis, and defense functions remain incompletely understood (127). In the zebrafish gut, resident macrophages are known to participate in the regulation of the microbiota (128). Additionally, these cells within the gut muscle layers interact with enteric neurons to coordinate smooth muscle contractions (85, 127).

In response to signals from the surrounding tissues, macrophages undergo molecular changes and exhibit different functional behaviors through a process known as macrophage polarization (129). Following polarization, macrophages can assume either the M1 type (classically activated macrophages), characterized by activation and the expression of pro-inflammatory modulators, or the M2 type (alternatively activated macrophages), characterized by high levels of anti-inflammatory mediators (130, 131). Macrophage polarization is believed to be induced by pathogens or their excreted-secreted molecules (129, 131).

It has been suggested that a successful acute inflammatory response leads to the elimination of infectious agents, followed by a resolution and repair phase facilitated by tissue-resident and recruited macrophages (132). *In vitro* stimulation of macrophages with pathogen-associated molecules like lipopolysaccharides or peptidoglycan results in increased production of oxygen radicals, pro-inflammatory chemokines and cytokines, as well as enhanced phagocytic activity (1). Macrophages express plasmalemma receptors, including toll-like receptors, scavenger receptors, and pathogen pattern recognition receptors (1). Furthermore, in addition to their phagocytic activity, macrophages function as antigen-presenting cells, binding antigens to T cells (133).

Accounts of fish macrophages and MAs against helminth infections have been reported (25, 134). At the site of inflammation, macrophages are exposed to dying cells and pro-inflammatory stimuli (135). The intestine harbors the largest pool of macrophages, responsible for maintaining mucosal homeostasis and epithelial renewal. Macrophages appear to be maintained in a steady state within the lamina propria of the fish intestine, protecting the mucosa against parasites/pathogens (12, 18) while also scavenging foreign debris and dead cells (136). In zebrafish experimentally infected with the pathogen *Streptococcus iniae*, neutrophils were found to produce leukotriene B4 (LTB4), which regulates macrophage aggregation (137).

Macrophages exhibited immunoreactivity to serotonin and i-NOS antibodies, displaying strong reactivity primarily within the outer cytoplasmic region (88). In the intestine of mullet *Chelon ramada* infected with the myxozoan *Myxobolus mugchelo*, a significant number of large and atypical intraepithelial macrophages were observed engulfing *M. mugchelo* spores and necrotic debris (88).

In the livers of fish *Gymnotus inaequilabiatus* harboring nematode larvae, the presence of macrophages and MAs was remarkable (73). Furthermore, in the swimbladder of European eels infected with the nematode *Anguillicoloides crassus*, a considerable number of macrophages and MCs were observed within the submucosal layer (17, 138).

### 2.5 Epithelioid cells

After infection, the extent of the subsequent host reaction can vary considerably, and each encysted parasite is often surrounded by granulomatous tissue (73). Fish granulomas are inflammatory focal points consisting of concentric layers of epithelioid cells (63) and various types of host immune cells, resembling mammalian granulomas closely (139, 140). The formation of granulomas in response to extra-intestinal parasites in fish has been extensively documented in the intestines and viscera (63, 141). Granulomas are chronic inflammatory lesions that often manifest as nodules (73) in one or multiple organs (142). Epithelioid cells derive their name from their morphological resemblance to epithelial cells (143). These cells are typically transformed macrophages, primarily responsible for phagocytosing foreign agents (144–146).

The inner layer of granulomas closest to the parasitic larva mainly consisted of dark necrotic epithelioid cells (72). Non-necrotic epithelioid cells formed desmosomes with each other (72) or with the fibroblasts. Epithelioid cells possess nuclei rich in euchromatin, and their cytoplasm contains numerous filaments, free ribosomes, and swollen mitochondria. In some cases, the epithelioid cells exhibited a foamy appearance. Granulomas have been observed surrounding encysted larvae of nematodes in the organs of various fish species (12, 63, 72, 147, 148), digenean larvae in tench organs (149), and cestodes in the liver of perch (150). In zebrafish granulomas caused by mycobacteria, the epithelioid cells exhibit elevated levels of E-cadherin, forming a closed-cell envelope around the pathogens (151). It has been hypothesized that such concentric layers of epithelioid cells could serve as a protective barrier for pathogens against the host’s immune response (151, 152), or they may “isolate” the pathogen and prevent damage to the host’s tissues (152).

### 2.6 Erythrocytes

Several studies have established the involvement of fish red blood cells in innate and adaptive immune processes, in addition to their role in gas exchange mechanisms (153). Unlike higher vertebrates, the erythrocytes of Osteichthyes are oval in shape, possess a nucleus, and rarely exhibit visible cytoplasmic organelles, likely due to hemoglobin storage (154).

Fish erythrocytes can modulate the expression of different sets of gene in response to stimuli (155, 156). They also produce antimicrobial peptides and cytokines (157, 158) and are involved in the elimination of pathogens associated with complement components (159). Similar to neutrophils and macrophages, fish erythrocytes can engulf micro-pathogens or molecular debris through erythrophagocytic processes (159, 160). Furthermore, these cells possess pattern pathogen recognition receptors, enabling them to function as antigen-presenting cells via major histocompatibility complex class II antigens (157).

Several studies have focused on the involvement of fish erythrocytes in immune processes, specifically concerning viruses (156, 159, 161), bacteria (160), and fungi (162). A recent review by Stosik et al. (153) provides insights into the function of fish erythrocytes in immunity against micro-pathogens. This evidence highlights the significance of these cells in host defense against pathogens (155). However, there is currently no information available regarding the potential role of these cells in metazoan infection of fish tissues/organs.

### 2.7 Rodlet cells

Rodlet cells (RCs) are pear-shaped cells characterized by a distinctive cortex, basal nucleus, and conspicuous typical inclusions called rodlets (163, 164). Rodlet cells are primarily found in the epithelial tissue of the intestine, gonads, swim bladder, skin, gills, heart, sensory organs, brain, thymus, liver, spleen, kidney, in freshwater and marine fish (164). For over 120 years, fish pathologists and histologists have debated the origins and functions of these enigmatic cells. The first review of RCs, published by Manera and Dezfuli (163), reported contrasting perspectives on the nature and function of RCs, along with several unresolved issues. The parasitic nature of RCs leaves many questions unanswered. For instance, why do these cells lack a specific tissue preference? If RCs are a type of protozoan parasite (Apicomplexa), it is challenging to explain why their number increases in fish infected with another protozoan (17, 164). Extensive literature on RCs as endogenous fish cells exists and continues to grow. Consequently, in investigations of numerous fish species, no evidence of inflammation in the surrounding tissue of RCs has been found. Moreover, RCs have been observed in neonates or very young laboratory-reared fish, embryos of viviparous teleosts, and newly hatched fish obtained under pathogen-free, quality-controlled conditions (165, 166). Some claims suggest that RCs are a type of inflammatory cell closely associated with other piscine inflammatory cells, such as MCs, mesothelial, and epithelioid cells (65). Additionally, RCs are considered a kind of secretory cell and proliferate in response to tissue injury or related factors (164, 167, 168). In the intestines of *A. anguilla* and *C. carpio*, RCs express immune molecular markers, including lysozyme and polysaccharides, such as α-N-acetyl-galactosamine (164, 168). Lysozyme, being an antimicrobial enzyme, has a significant role in the innate immunity of fish and its presence in RCs strengthens the defensive role of these cells against pathogens (164). Indeed, α-N-acetyl-galactosamine was detected in MCs of different species of fish (59, 164, 168), it was suggested that its residues in the carbohydrate backbone were involved in the protection of the mucosae from microorganisms (35).

Records concerning the role of RCs as immune effector cells have primarily focused on their mobilization and recruitment in response to microparasites such as viruses (169), bacteria (168, 170), protozoans (17, 164, 171), and myxosporeans (88, 167, 171–173). In fish hosting macroparasites, the presence of an increased number of RCs, particularly at the site of infection (12), provides further evidence of their defensive function as part of the innate immune system (12, 17, 60, 174). As previously mentioned, the initial review on RCs was published 18 years ago, and a subsequent edition was necessary to update the current understanding of the origin, structure, and function of these intriguing fish cells (164).

Rodlet cells are unique cells exclusively found in teleosts. However, two Egyptian research groups (175, 176) observed a kind of cells in the alimentary canals of two bird species and named them RCs. These bird cells bear minimal resemblance to fish RCs, and the authors did not provide sufficient compelling data to support their interpretations. Notably, RCs have not been reported in elasmobranch tissues, which are much closer relatives to teleosts than birds. This observation raises doubts regarding the existence of RCs in birds, and parsimony leads us to suspect that the two bird species possess RCs while elasmobranch fish lack them.

### 2.8 Parasite-host counter-adaptation, who is calling the shots?

Due to their elongated body plan, helminths are macroparasites that cannot be ingested by host phagocytes such as macrophages and neutrophils (177). Helminths are highly successful pathogens primarily due to their evolution of potent and diverse immune subversion strategies, which enable them to evade host immune responses effectively (178–180). Their remarkable co-evolution with the host’s immune system allows helminths to infect multicellular species across various geographical environments (181). A substantial portion of our understanding regarding the structure, function, and regulation of host immune responses and the excretory-secretory (ES) products of parasites has been derived from studies on mammal-helminth systems (177, 182, 183). Helminth secretomes encompass a multitude of potential immunomodulators, and the molecular and functional diversity of these entities at the host-parasite interface have gained increasing recognition (180, 183, 184). Consequently, these molecules play an essential role in the survival of the parasite within the host (177, 179, 183, 185). Helminth ES products comprise extracellular vesicles (EVs) that contain proteins, lipids, and RNAs, serving as carriers for immune modulators targeted at specific cell types (179, 186, 187). EVs, which are membrane-enclosed nanoparticles, are a common feature of parasite secretion across a wide range of species; further details can be found in (188). Two types of EVs have been proposed based on their size and biogenesis (188). Numerous studies have been published on the ES products of human helminths, particularly focusing on nematodes (e.g., 183, 189, 190), and lesser on cestodes (191–193), and trematodes (194, 195).

Four taxa, namely trematodes (flukes), cestodes (tapeworms), nematodes (roundworms), and acanthocephalans (spiny-headed worms), encompass the helminths found in aquatic vertebrates. Similar to helminths infecting terrestrial vertebrates, helminths of teleost fish have developed strategies to manipulate and evade host immune responses. These strategies involve the release of extracellular vesicles by parasites (196–198). Several studies have investigated the effects of helminth ES products on piscine leukocytes (e.g., 199–201). Experimental *in vivo* infections have demonstrated that *Schistocephalus solidus* (Cestoda) can alter the cellular immune responses of its fish second intermediate host (202), and similar findings have been reported in three salmonid species infected with the nematode *Anisakis simplex* (200, 203).

In recent years, the characterization of extracellular vesicles from zoonotic nematode species, such as *Anisakis* spp., has garnered the attention of several authors (196, 198, 204, 205). Regarding the *Anisakis simplex*-rainbow trout system, the ES products of the nematode had an immune depressive effect; accordingly, worm enzymes reduced the fish immune response and increased parasite survival (200). Over 40 years of direct evidence on the occurrence and stability of helminths in numerous fish species suggest that not all fish species are capable of mounting effective defenses against helminths. Furthermore, in four different taxa of endoparasitic helminth species in fish at the host-parasite interface regions, no extracellular vesicles containing tegumental secretions of the worms were observed (95). It appears that in high-intensity liver infections of *Gymnotus inaequilabiatus* and *Micromesistius poutassou* with the nematodes *Brevimulticaecum* sp. and *Anisakis simplex*, respectively, organ functions are likely to be severely compromised (73, 148). However, it should be noted that both of these species were alive before necropsy.

Invasion of tissues can have more serious pathological implications, depending on factors such as worm size, infection intensity, and parasite stage (206). However, there are very few documented cases of wild fish eliminating helminths. Instances of helminth destruction have only been observed in the liver of *Lota lota* and *Perca fluviatilis*, where *Triaenophorus nodulosus* larvae were affected (207).

Insights from various areas of parasitology research, including immunoparasitology and pharmacology, can drive the development of new methods aimed at altering host-parasite interactions through the suppression of parasite ES products, with the goal of developing vaccines (208), novel anthelmintic strategies (180, 187), and exploring therapeutic potential (204). These studies may provide valuable insights into the question of “Who is calling the shots?” in fish helminth infections, but the mechanisms of immunoavoidance and immunosuppression in these parasites remain unclear.

## 3 Concluding remarks

Global fish consumption has witnessed an increase in recent years, and this upward trend is expected to persist (209). However, the presence of parasites poses a substantial threat to both wild-caught and cultured fish. Parasitic infections are highly prevalent in wild fish populations, and the rising popularity of consuming raw and smoked fish necessitates diligent parasite monitoring to mitigate the risk of disease transmission. Within the realm of fish mariculture and aquaculture, metazoan parasites stand out as particularly pathogenic organisms capable of causing zoonotic infections in consumers. To date, no commercial vaccines have been developed to combat parasitic diseases in fish. Hence, it is imperative that we expand our understanding of basic biology to devise sustainable strategies for controlling fish parasites. A more comprehensive understanding of fish defense mechanisms will serve as a foundation for the development of health management tools, thereby facilitating the growth of sustainable aquaculture and mariculture industries.

Both protozoan and metazoan parasites encounter the cellular and humoral components of fish immune systems, resulting from the co-evolution of the immune response of the host and the evasive mechanisms employed by the parasite. While significant progress has been made in elucidating the molecular mechanisms underlying immunomodulation by various ES proteins and other products generated by mammalian helminths, our understanding of the occurrence and effects of helminth ES proteins on fish immune systems is still in its nascent stages. Further investigations are required to unravel the relationship between the fish immune system and protozoan and metazoan parasites. Additionally, immunohistochemical studies can contribute to our comprehension of the mechanisms and interactions involving fish innate immune cells and parasites. The application of molecular and immunopathological approaches to fish-parasite systems will enhance our understanding of fish pathology and provide insights into immune mechanisms in fish. We hope that the data presented in this article will inspire further research on the interactions between fish innate immune cells and parasites.

## Author contributions

BSD: Conceptualization (lead), writing the original draft (lead). ML: editing (supporting). AC: editing (supporting). LG: Writing-review and editing. GB: Conceptualization (equal), writing-original draft (lead). All the authors contributed to the manuscript and approved the submitted version.
